# *In Silico* Prediction for Intestinal Absorption and Brain Penetration of Chemical Pesticides in Humans

**DOI:** 10.3390/ijerph14070708

**Published:** 2017-06-30

**Authors:** Lisa Chedik, Dominique Mias-Lucquin, Arnaud Bruyere, Olivier Fardel

**Affiliations:** 1 Institut de Recherche en Santé, Environnement et Travail (IRSET), UMR INSERM U1085, Faculté de Pharmacie, Université de Rennes 1, 2 Avenue du Pr Léon Bernard, 35043 Rennes, France; lisa.chedik@univ-rennes1.fr (L.C.); arnaud.bruyere@univ-rennes1.fr (A.B.); 2 Institut de Génétique et Développement de Rennes, UMR CNRS 6290, Université de Rennes 1, 35043 Rennes, France; dominique.mias-lucquin@univ-rennes1.fr; 3 Pôle Biologie, Centre Hospitalier Universitaire, 2 rue Henri le Guilloux, 35033 Rennes, France

**Keywords:** pesticides, toxicokinetics, intestinal absorption, brain permeation, toxicity, *in silico* method, prediction

## Abstract

Intestinal absorption and brain permeation constitute key parameters of toxicokinetics for pesticides, conditioning their toxicity, including neurotoxicity. However, they remain poorly characterized in humans. The present study was therefore designed to evaluate human intestine and brain permeation for a large set of pesticides (*n* = 338) belonging to various chemical classes, using an *in silico* graphical BOILED-Egg/SwissADME online method based on lipophilicity and polarity that was initially developed for drugs. A high percentage of the pesticides (81.4%) was predicted to exhibit high intestinal absorption, with a high accuracy (96%), whereas a lower, but substantial, percentage (38.5%) displayed brain permeation. Among the pesticide classes, organochlorines (*n* = 30) constitute the class with the lowest percentage of intestine-permeant members (40%), whereas that of the organophosphorus compounds (*n* = 99) has the lowest percentage of brain-permeant chemicals (9%). The predictions of the permeations for the pesticides were additionally shown to be significantly associated with various molecular descriptors well-known to discriminate between permeant and non-permeant drugs. Overall, our *in silico* data suggest that human exposure to pesticides through the oral way is likely to result in an intake of these dietary contaminants for most of them and brain permeation for some of them, thus supporting the idea that they have toxic effects on human health, including neurotoxic effects.

## 1. Introduction

Chemical pesticides constitute an important group of substances, exhibiting notably insecticide, rodenticide, fungicide, nematicide, or herbicide properties, which are used to control and repel pests in different fields. They can be grouped into various chemical families, including the pyrethroids, organochlorines, carbamates, organophosphorus compounds, triazines, and neonicotinoids. Due to their large use for agricultural, industrial, or domestic purposes, and also due to the strong persistence of some of them, pesticides appear as widely-distributed environmental pollutants [1]. Humans are consequently highly exposed to these chemicals—notably by an oral way—through the consumption of food or water contaminated by pesticides [2,3,4]. This is probably a major health issue, owing to the well-established toxicity of pesticides, which can trigger or favor various diseases including cancers and neurodegenerative, metabolic, reproductive, or developmental pathologies [5,6]. Such adverse effects are thought to reflect specific toxicodynamic properties of pesticides, such as agonistic or antagonistic effects towards endocrine receptors, genotoxicity, the blockage of ion channels, the inhibition of enzymes such as acetylcholinesterase, the impairment of the redox balance, or oxidative stress generation [7,8,9].

Before exerting toxicodynamic effects, pesticides have however to be absorbed by exposed humans, notably at the gastro-intestinal level in response to oral exposure; neurotoxic ones have additionally to cross the blood-brain barrier [10]. Such a consideration for intestinal absorption and brain distribution is also a major concern for drugs, notably during their industrial development [11,12]. In this context, the passage of drugs across the intestinal barrier and the blood-brain barrier is usually extensively characterized using *in vitro* cellular models, animal experimentation, and clinical human pharmacokinetics studies before marketing authorization [13,14,15]. The intestinal absorption and brain disposition of drugs can moreover be *in silico* predicted with good accuracy, using various computer models [16,17,18,19]. Among them, SwissADME is a recent free web tool designed for predicting pharmacokinetics parameters [20]. It notably concomitantly evaluates the intestinal and brain permeation of drugs through an accurate predictive model that works by computing the lipophilicity and polarity of drugs with good accuracies (83–93% for intestinal absorption and 90% for brain permeation [21]). This physicochemical descriptors-based method, termed the BOILED-Egg method, permits a clear and informative graphical representation of the data, and can be applied in a variety of settings, from the filtering of chemical libraries at the early steps of drug discovery, to the evaluation of drug candidates for development [21].

In contrast to drugs, pesticides are very poorly characterized with respect to their intestinal and brain passage in humans. For a very limited number of them, intestinal absorption has been characterized from pharmacokinetics studies performed with human volunteers [22]. Some information may additionally be extrapolated from animal studies or *in vitro* studies [23]; however, the animal versus human bioavailability of chemicals is known to be poorly correlated [24], making it difficult to precisely and unambiguously predict the human absorption of pesticides from animal data. The intestinal and brain permeations of pesticides are nevertheless important to evaluate, in order to fully apprehend their potential toxicity, as discussed above. In this context, the use of *in silico* tools may be considered a valuable and robust approach. Indeed, the great number and variety of pesticides which have to be evaluated precludes the use of cost-effective and labor-consuming classical pharmacokinetics *in vitro* or *in vivo* methods. Clinical pharmacokinetics studies in human subjects with pesticides are moreover now hampered by appropriate ethical considerations [25,26]. The present study was therefore designed to evaluate *in silico* the intestinal absorption and brain permeation of 338 pesticides belonging to various chemical classes, using the SwissADME web tool.

## 2. Materials and Methods

### 2.1. Pesticide Selection

The complete list of pesticides (*n* = 338) included in the study is shown in Table S1, while the list of oxon metabolites of organophosphorus pesticides (*n* = 15) is provided in Table S2. The selection of pesticides was driven by retaining the main members of various major chemical classes of pesticides, i.e., the organochlorines (*n* = 30), the organophosphorus compounds (*n* = 99), the pyrethroids (*n* = 42), the carbamates (*n* = 74), and the triazines (*n* = 31); miscellaneous pesticides (*n* = 62) belonging to various additional classes of pesticides, such as the neonicotinoids, were also included. Most if not all of these chemicals are included in pesticide and/or chemical data bases, such as the Hazardous Substance Data Bank (HSDB), a Toxicology data file on the U.S. National Library of Medicine’s (NLM) Toxicology Data Network (TOXNET) (Bethesda, MD, USA), which is freely available online [27]. They are referenced by organizations devoted to pesticide concerns, such as, for example, the National Pesticide Information Center (Corvallis, OR, USA), a collaboration between Oregon State University and the U.S. Environmental Protection Agency to provide objective, science-based, and accessible online information about pesticides [28]. They commonly exhibit insecticide, fungicide, or herbicide properties, and correspond to registered products currently in use or banned products due to toxicity issues. Some pesticide metabolites or degradation by-products as well as the pesticide impurities dibenzo-para-dioxin and 2,3,7,8-tetrachlorodibenzo-p-dioxin (TCDD) [29] were also included in the list of studied chemicals.

### 2.2. In Silico Evaluation of Intestine and Brain Permeation

A Simplified Molecular Input Line Entry System (SMILES) was collected online for each pesticide of the Table S1 and also for the oxon metabolites of the organophosphorus pesticides (Table S2) through interrogating the PubChem data base (U.S. National Library of Medicine) [30]; they are indicated in Table S1 for each pesticide and in Table S2 for each oxon organophosphorus pesticide. The list of SMILES was then entered in the online SwissADME web tool for generating a prediction of the gastrointestinal absorption (high or low) and brain penetration (yes or no). These predictions were done using the BOILED-Egg method, which corresponds to a descriptive graphical approach discriminating between intestinal well-absorbed and intestinal poorly absorbed molecules, and between brain-permeant and brain non-permeant molecules. This method is primarily based on two parameters: (1) the lipophilicity of chemicals, evaluated from partition-coefficient (P) by a LogP value calculated according to the Wildman–Crippen method (WLogP), and (2) their polarity, determined by a calculated topological polar surface area (tPSA) value. A prior analysis of human absorption and brain permeation databases for drugs has allowed us to delineate two ellipsoidal regions (one for intestine permeation and one for brain permeation, which are not mutually exclusive) on the graph WLogP versus tPSA [21]. The compounds included in these ellipses are predicted to display high intestinal absorption, i.e., the fraction absorbed (Fa) is ≥0.30, with Fa equal to the amount of absorbed chemical/amount of ingested chemical, or brain permeation*,* i.e., the brain-blood concentration ratio is ≥1 (the LogBB, defined as the logarithm of the ratio of the drug concentration in the brain versus that in the blood, is therefore ≥0) [20]. Such predictions have been previously performed with good accuracy (around 90%) for drugs [21]. The ellipsoidal regions defining the intestine and brain permeation were drawn as initially reported in [21] using the software R (R Foundation for Statistical Computing, Vienna, Austria) [31]); the specific R code used is shown in Appendix A.

### 2.3. Association of Pesticide Physicochemical Parameters and Predicted Permeation across Intestinal or Blood-Brain Barriers

Physicochemical parameters such as molecular weight, the number of heavy atoms, the number of aromatic heavy atoms, the number of rotatable bonds, the number of H-bond acceptors, the number of H-bond donors, the fraction of carbon bond saturation (Csp3), i.e., the number of sp3 hybridized carbons/total carbon count, the solubility (S) parameter LogS (Silicos-IT), the lipophilicity parameter LogP predicted using the additive XLogP3 method [32], and the molar refractivity were given for each pesticide by the online SwissADME web tool. Additional basic molecular descriptors such as the mean atomic van der Waals volume, the mean atomic polarizability, and the number of nitrogen and oxygen atoms were obtained using the Dragon 7.0 software (Talete, Milano, Italy), as previously described in [33]. The values of each of these parameters were next compared between the predicted intestine or brain-permeant and non-permeant pesticides by the Student’s *t* test, in order to determine the nature of the physicochemical parameters that may be associated with the predicted intestine or brain permeation of the pesticides. The criterion of significance was *p* < 0.05.

### 2.4. Confrontation of Predicted and Measured Human Intestinal Absorption Values for Some Pesticides

The curation of measured human intestinal absorption for pesticides was performed through searching available pharmacokinetics studies related to the oral administration of pesticides in human subjects. For this purpose, PubMed (US National Library of Medicine) [34] and HSDB [27] were interrogated online for each pesticide included in the study. The Fa values were directly collected when they were provided in human pharmacokinetics studies, or were estimated from the excreted fraction in urine of the ingested dose of pesticide. When the Fa values were given as a range of values for one ingested dose, the median value was retained. When different Fa values were reported for one pesticide in various studies and/or for different ingested doses, the mean value was retained. The pesticides were considered as exhibiting high or low measured intestinal absorption when the measured experimental Fa was ≥0.30 or <0.30, respectively. The measured and predicted intestinal absorption was then compared for each considered pesticide. Accuracy of the prediction was finally determined using the following equation:

$$Accuracy\;\left(\%\right)=\frac{Total\;number\;of\;pesticides\;with\;identical\;predicted\;and\;measured\;intestinal\;absorption}{Total\;number\;of\;studied\;pesticides}\;\times 100$$

## 3. Results

### 3.1. Prediction of Intestinal and Brain Permeation of Pesticides

**Figure 1:**
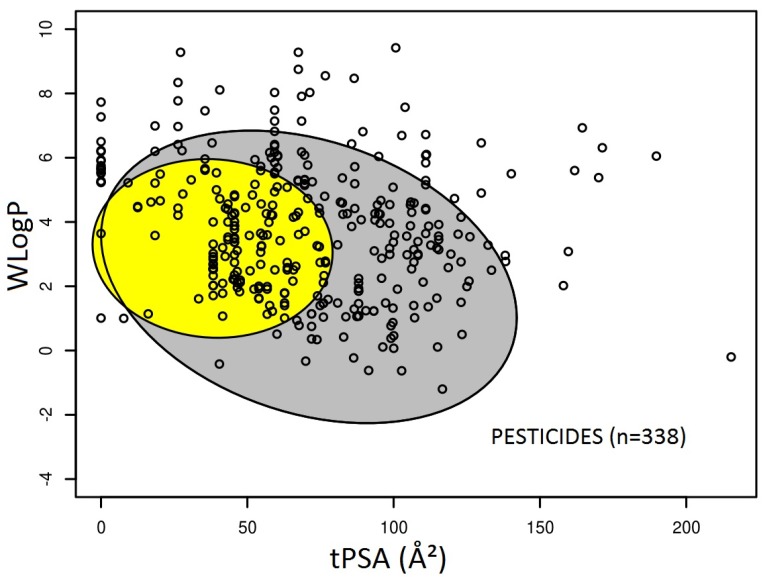
Graphical distribution of pesticides using the BOILED-Egg predictive model for intestine and brain permeation. Each pesticide corresponds to a small black circle. The grey region is the physicochemical space of pesticides (*n* = 275) predicted to exhibit high intestinal absorption, and the yellow region is the physicochemical space of pesticides (*n* = 130) predicted to permeate the brain. Abbreviation: topological polar surface area (tPSA). WLogP: LogP value calculated according to the Wildman–Crippen method.

The predictions of intestinal absorption and brain permeation were determined using the tPSA/WLogP-based graphical BOILED-Egg method included in the SwissADME tool and are indicated for each pesticide in Table S1. When considering the total number of pesticides (*n* = 338), 275 pesticides (81.4% of pesticides) exhibited a predicted high intestinal absorption, as demonstrated by their location inside the grey ellipse defining intestinal absorption (Figure 1). A lower number of pesticides (*n* = 130, representing 38.5% of pesticides) is comprised inside the yellow ellipse corresponding to brain-permeant molecules (Figure 1), and can therefore be predicted to show brain permeation. Essentially, all of the pesticides predicted to enter the brain also displayed a predicted high intestinal absorption. This was expected, owing to the fact that the brain permeation-related ellipse defined by the BOILED-Egg method is almost entirely comprised into the intestine permeation ellipse (Figure 1). Only the organochlorine lindane was predicted to display low intestinal absorption, but high brain permeation (Table S1).

**Figure 2:**
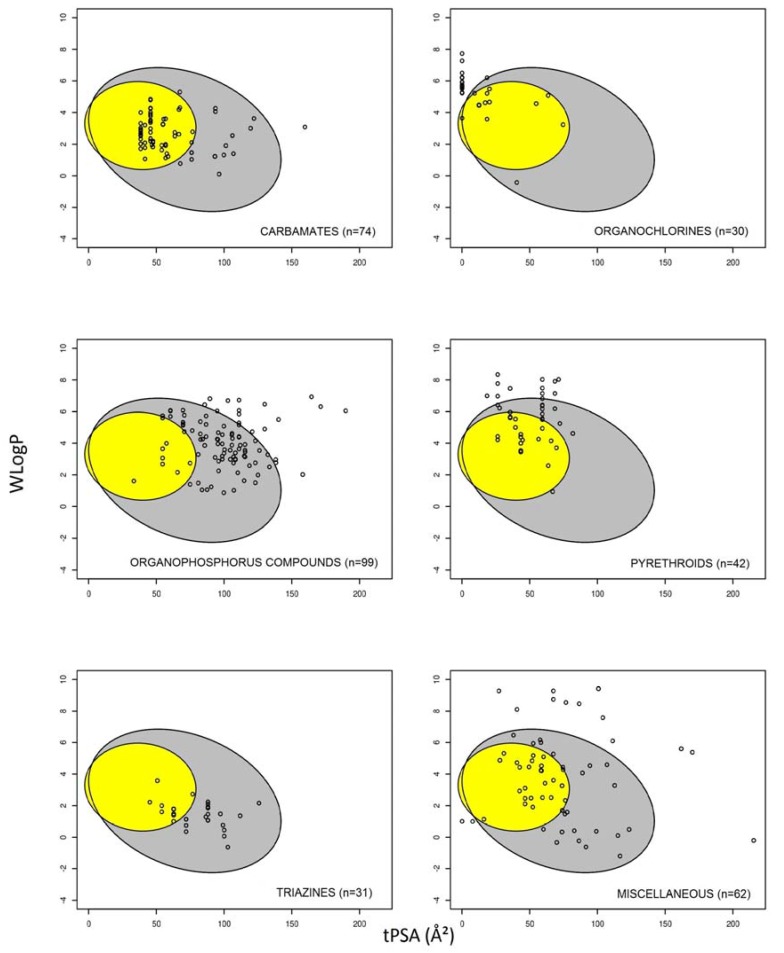
Graphical distribution of pesticides according to chemical classes using the BOILED-Egg predictive model for intestine and brain permeation. Each member of a chemical pesticide class corresponds to a small black circle. The grey region is the physicochemical space of pesticides predicted to exhibit high intestinal absorption, and the yellow region is the physicochemical space of pesticides predicted to permeate the brain. tPSA: Topological polar surface area; WLogP: LogP value calculated according to the Wildman–Crippen method.

**Figure 3:**
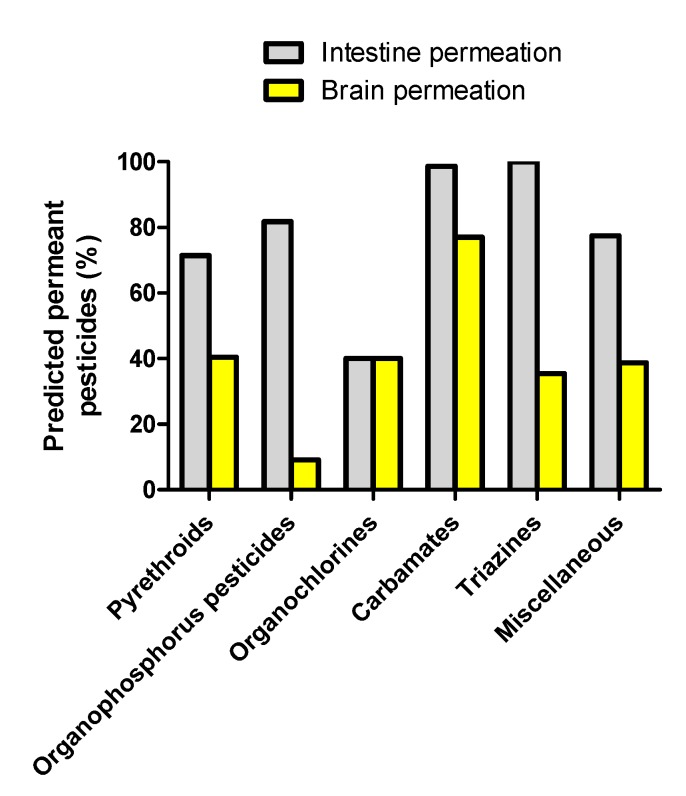
Percentage of predicted intestine- and brain-permeant molecules for the main chemical classes of pesticides.

The graphical prediction of intestine absorption and brain permeation by the pesticide classes is shown in Figure 2. The percentage of predicted intestine-permeant molecules was very high for the triazines (100% permeant) and the carbamates (99% permeant); high for the organophosphorus pesticides (82% permeant), the miscellaneous pesticides (77% permeant), and the pyrethroids (71% permeant); and lower for the organochlorines (40% permeant) (Figure 3). Organochlorines such as aldrin, chlordane, dichlorodiphenyltrichloroethane (DDT), heptachlor, and lindane, which failed to successfully meet the criteria for high intestinal absorption, were those with a null value for tPSA and a high WLogP value (Table S1 and Figure 2), indicating that they correspond to highly lipophilic non-polar chemicals. With respect to the prediction of brain permeation, the chemical class with the highest percentage of permeant pesticides was the carbamates (77% permeant), followed by the pyrethroids (41% permeant), the organochlorines (40%), the miscellaneous pesticides (39% permeant), the triazines (36% permeant), and the organophosphorus compounds (9% permeant) (Figure 3). Pesticides from the carbamate, triazine and organophosphorus pesticide classes, which were predicted to be brain non-permeant, display globally higher tPSA values than their permeant counterparts (Figure 2), thus suggesting that they correspond to more polar chemicals.

Some organophosphorus pesticides require metabolic bioactivation to be maximally active [35,36]. Organophosphorus oxon metabolites, formed notably upon the action of hepatic cytochromes P-450, have thus been shown to exert potent irreversible anti-acetylcholinesterase activity and to cause neurotoxicity [37]. The passage of such oxon metabolites across the blood-brain barrier is therefore important to consider, in addition to that of the parent molecules. The prediction of brain permeation was therefore performed for oxon metabolites from 15 different organophosphorus pesticides using the BOILED-Egg method. As shown in Table 1, none of the parent organophosphorus compound was predicted to cross the blood-brain barrier. By contrast, three oxon metabolites were predicted to do it (Table 1), resulting however in a rather low percentage of brain-permeant oxon metabolites (20%).

### 3.2. Pesticide Physicochemical Parameters Associated with the Prediction of Intestinal or Brain Permeation

The values of different physicochemical parameters were first compared between the predicted intestine permeant and non-permeant pesticides. As indicated in Table 2, various parameters such as molecular weight, mean atomic van der Waals volume, mean atomic polarizability, number of heavy atoms, LogS, XLogP3, and molar refractivity were found to discriminate between predicted intestine-permeant and non-permeant pesticides with a high level of significance (*p* < 0.0001). The predicted intestine-permeant pesticides have thus notably lower molecular weight, mean atomic van der Waals volume, mean atomic polarizability, XLogP3-related lipophilicity, and molar refractivity than predicted non-permeant counterparts, whereas they exhibit higher solubility. Other parameters, such as the number of rotatable bonds and the number of H-bond acceptors, also differentiate permeant and non–permeant pesticides, but with a more moderate level of significance, whereas the parameters fraction Csp3, number of aromatic heavy atoms, and number of H-bond donors fail to do it (Table 2). When these molecular descriptors were applied to one particular class of pesticides, such as the organochlorine class, which approximately comprises similar proportions of predicted intestine-permeant and non-permeant chemicals (Figure 3), only XLogP3, LogS, the number of H-bond acceptors, and the number of H-bond donors reached significant levels (Figure S1). The parameter tPSA also highly discriminates intestine-permeant and non-permeant organochlorines (Figure S1), whereas it fails to do it for the whole set of pesticides (Table 2).

With respect to the prediction of brain permeation for pesticides, the molecular weight, the number of rotatable bonds, and the number of H-bond acceptors, as well as tPSA, constitute discriminative physicochemical parameters with a high level of significance (*p* < 0.0001) (Table 3). The predicted brain-permeant pesticides display thus a lower molecular weight, number of rotatable bonds, number of H-bond acceptors, and tPSA than their non-permeant counterparts. The number of heavy atoms and molar refractivity also differentiate predicted permeant and non-permeant pesticides, but with a lower level of significance (Table 3). The number of nitrogen and oxygen atoms, which is recognized as one of the main parameters to consider for brain penetration [38], was additionally highly significantly (*p* < 0.0001) lower for pesticides predicted to be brain-permeant than for their brain non-permeant counterparts.

### 3.3. Confrontation of Predicted and Measured Human Intestinal Absorption for Some Pesticides

The prediction of human intestinal absorption determined from the tPSA/WLogP-based graphical BOILED-Egg method was finally compared with the measured intestinal absorption for a limited number of pesticides (*n* = 25) belonging to various chemical classes of pesticides, and for which human pharmacokinetics data are available. The threshold retained for predicted and measured high intestinal absorption was the same, i.e., Fa ≥ 0.30. As shown in Table 4, the prediction for almost all of the considered pesticides (*n* = 24) was confirmed with human experimental data. The accuracy of the prediction was consequently found to correspond to 96%.

## 4. Discussion

Human intestinal absorption and human brain distribution correspond to key steps of pesticide toxicokinetics, but remain poorly characterized. In the present study, we have used a recent and original web tool, i.e., SwissADME, previously developed and validated for drugs [20], for investigating *in silico* the human intestine and brain permeation of 338 various pesticides belonging to the main chemical classes of pesticides. Our results showed that a large proportion of pesticides (more than 80%) is predicted to be highly absorbed by the human gastro-intestinal tract. This supports the idea that human exposure to pesticides through the oral way, which commonly occurs via the ingestion of contaminated food or water, is likely to result in an intake of these environmental contaminants. This may ultimately favor their deleterious effects towards human health, including endocrine disruption and carcinogenic effects [5]. In addition, a notable, although minor, proportion of pesticides (around 38%) is predicted to permeate the brain, which may support the well-established idea that pesticides have neurotoxic effects [39].

The prediction of intestinal absorption of pesticides performed with the SwissADME/BOILED-egg method was interestingly validated with absorption data from human toxicokinetics studies, with an accuracy of 96%. Even if the available measured data from the human studies are unfortunately limited (*n* = 25), and rather unbalanced, i.e., most of them (*n* = 23) reporting high intestinal absorption of pesticides (Table 4), this validation is worth noting. A similar high accuracy (83–93%) has also been reported for the prediction of human intestine absorption by the SwissADME tool for a large data set of drugs [21]. In addition, pesticides predicted to display high intestinal permeation exhibit a profile of physicochemical parameters, i.e., reduced molecular weight, van der Waals volume, and polarizability, and increased solubility, which is well-known to favor the high intestinal absorption of drugs and chemicals [70,71,72]. This supports the relevance of the *in silico* prediction. In particular, the size of the drug molecule affects absorption. Smaller molecular weight drugs are consequently absorbed better compared to larger ones [73]. Indeed, as molecular size increases, a larger cavity must be formed in water to be soluble. Increasing the size also impedes passive diffusion through the tightly packed aliphatic side chains of the lipid bilayer membrane. Moderately lipophilic drugs, i.e., drugs with LogP < 5, are additionally more absorbed than highly lipophilic drugs [74]; in agreement with this rule, *in silico* predicted high intestine-permeant pesticides have significantly lower LogP (estimated notably by the XlogP3 value in the present study) than their non-permeant counterparts. It should however be kept in mind that each physicochemical parameter, when separately considered, may fail to unambiguously predict intestine permeation, thus highlighting the relevance of combining two parameters as is done in the BOILED-Egg method. Finally, the *in vitro* measurement of transport across human intestinal Caco-2 cells for some pesticides, such as the neonicotinoids acetamiprid and imidacloprid, have revealed high apparent permeability coefficients [75,76] consistent with *in vivo* absorption with 100% efficiency, and thus with the *in silico* prediction of high absorption for these two pesticides (Table S1).

The prediction of brain permeation for pesticides cannot, unfortunately, be validated by human experimental pharmacokinetics data, owing to the quasi-absence of such data in the scientific literature and pesticide database. Nevertheless, the fact that the BOILED-Egg method predicts drug transfer across the blood-brain barrier with high accuracy [21] supports the relevance of this method for other chemicals such as pesticides. In addition, some physicochemical parameters significantly associated with the prediction of brain permeation for pesticides, such as a reduced tPSA and the number of nitrogen and oxygen atoms, are well-established factors favoring the permeation of drugs into the brain. Indeed, drugs entering the brain typically have a tPSA value of less than 60–70 Å^2^ [77] and a number of nitrogen and oxygen atoms less than or equal to five [38]. *In silico* predictions of brain passage were moreover supported by *in vitro* or *in vivo* data for some pesticides. The pyrethroid deltamethrin, predicted to be brain non-permeant (Table S1), has thus been shown to accumulate relatively poorly in human brain endothelial hCMEC/D3 cells [78]; whereas paraquat, also predicted to be brain non-permeant (Table S1), is excluded by the blood-brain barrier in the primate rhesus macaque [79].

Even if most of the pesticides are predicted to exhibit high intestine absorption, some of them, notably the organochlorines aldrin, chlordane, DDT, heptachlor, and lindane, are predicted to display low intestine absorption. Such intestine non-permeant organochlorines are highly lipophilic non-polar chemicals; they do not comprise H-bond acceptors or donors, in contrast to intestine-permeant organochlorines (Figure S1). This lack of potential H-bonds for the non-permeant organochlorines is consistent with their low solubility, probably contributing to their predicted poor intestinal absorption. It is noteworthy that the marked lipophilicity of the organochlorines also results in long-term storage in fat tissue and low excretion [80]. Therefore, the organochlorines, although some of them poorly absorbed, may nevertheless accumulate in an organism. These persistent organic pollutants may consequently reach cellular levels for which toxic effects, notably metabolic or endocrine effects, are suspected to occur [81,82]. In addition, the fact that the dietary intake of lipophilic organic chemicals such as organochlorines may occur primarily via adsorption to mixed dietary lipid micelles has to be considered [83,84]. Such a process may notably contribute to the intestine permeation of TCDD, thus explaining the discordance between the high measured intestine absorption of this organochlorine [51] and the prediction of low absorption by the BOILED-Egg method (Table 4).

It is noteworthy that the percentage of brain-permeant pesticides (less than 40%) is approximately twice as low as that of their intestine-permeant counterparts (more than 80%). This probably reflects the fact that the physicochemical criteria for entering the brain are more stringent than those required for intestinal absorption. In particular, concerning polarity, the tPSA value has to be ideally less than 60–70 Å^2^ for brain permeation, whereas the tPSA threshold is higher, i.e., 120 Å^2^, for oral absorption [77]. Organophosphorus pesticides are consequently predicted, for most of them, to be not brain-permeant, owing to their relatively high tPSA values (Figure 2); the bioactive metabolites of organophosphorus compounds, such as oxons-related molecules, are also rather poorly brain-permeant (Table 1). Similarly, the carbamates and triazines exhibiting substantial tPSA values are predicted to not cross the blood-brain barrier (Figure 2). It should be however kept in mind that the organophosphorus pesticides are suspected to cause neurotoxicity, including delayed neuropathies and long-term effects [85,86], which may appear as contradictory with the fact that they are predicted to be not brain-permeant. Such an apparent discrepancy may be due to the threshold retained for predicting a brain non-permeant chemical by the BOILED-egg method, i.e., LogBB < 0, whereas other studies have retained a less stringent threshold (LogBB < −1) for compounds poorly distributed in the brain [87,88,89]. In this context, it may be hypothesized that at least some of the organophosphorus pesticides can in fact partly access the brain, with LogBB values comprised between −1 and 0 (−1 < LogBB < 0), which may be compatible with their neurotoxicity. The fact that a LogBB equal to −0.09 has been measured for the organosphosphorus parathion in pigs probably supports this hypothesis [90]. Besides, organophosphorus pesticides such as chlorpyrifos and malathion directly alter the functional integrity of the blood-brain barrier [91,92,93]. Such a disruption of this blood-tissue barrier may secondarily result in the enhanced passage of organophosphorus pesticides in the central nervous system.

It is noteworthy that the BOILED-Egg method-based prediction of drug passage across the intestine and the blood-brain barrier does not directly take into account the potential implication of the influx or efflux of drug transporters [21]. However, there is now increasing evidence that such membrane transporters may interact with various pesticides [94], including organophosphorus compounds, organochlorines, paraquat, and pyrethroids [33,95,96,97,98]. In particular, the putative transport of pesticides at the intestinal level by ATP-binding cassette (ABC) efflux transporters such as P-glycoprotein (*ABCB1*) and the breast cancer resistance protein (BCRP*, ABCG2*) has probably to be taken into account [99]. Indeed, the low doses of pesticides contained in food are unlikely to saturate these efflux pumps, in contrast to the high doses of administrated drugs. The P-glycoprotein and/or BCRP-mediated efflux of pesticides at the apical pole of intestinal cells may consequently efficiently prevent their absorption by actively repelling them into the lumen of the intestine. Solute carrier (SLC) transporters acting as influx transporters may also have to be considered for the intestinal transport of some pyrethroids [100].

If intestinal absorption and brain penetration constitute key steps of pesticide toxicokinetics in response to oral exposure, other parameters have to be taken into account for more globally considering pesticide disposition in the human organism. This is notably the case for metabolism and/or excretion processes, which may occur rapidly and extensively, notably for first-pass hepatic metabolism, thus resulting in the relatively short half-lives of some pesticides. This may concern pyrethroids and organophosphorus insecticides, which are subjected to many metabolic pathways mediated by various xenobiotic metabolizing enzymes [101,102]. By contrast, organochlorine pesticides are thought to be less metabolized, and therefore appear to be more stable in the organism. Storage in some compartments, such as adipose tissue, may additionally play a major role in the toxicokinetics of pesticides, notably for the most lipophilic of them, such as the organochlorines [103], as discussed above. Binding to proteins, including plasma proteins, is also a key parameter to consider, as only the free fraction of pesticides is presumed to exert toxicodynamic effects and to be handled by pharmacokinetics effectors such as drug transporters and drug metabolizing enzymes. The chirality of pesticides may also have to be taken into account, as reported for drugs [104]. Entero-hepatic circulation, which occurs for some pesticides such as the carbamates and organochlorines [103,105], may additionally deserve attention. Moreover, the formulation of pesticides may constitute a feature influencing their intestinal absorption, notably for the organosphosphorus insecticides [52,106]. The fact that humans are usually exposed to a mixture of pesticides [107] may also affect their intestinal absorption, owing to possible pesticide–pesticide interactions with respect to drug transporters, as already described for drug–drug interactions [108]. Finally, the nature of the diet may interfere with the passage of pesticides across the intestinal barrier, as previously shown for the intestinal absorption of drugs [109].

Computer-based methods based on molecular descriptors such as lipophilicity and polarity, initially developed for drugs [16], may be useful for predicting not only pesticide intestine and brain permeation, as described in the present study, but also for evaluating other pharmacokinetics parameters such as metabolism and clearance, as well as the biological effects of pesticides. More globally, *in silico* methods and computational systems biology probably represent promising ways for assessing pesticide toxicity and risk, notably in humans [110,111,112,113]. However, further studies are required to fully validate *in silico* approaches, notably with respect to the realistic conditions of environmental exposure to pesticides, i.e., low and/or chronic doses of mixtures of pesticides. In particular, *in silico* methods are likely to be compared with other non-animal approaches, such as *in vitro* methods, for human toxicokinetics in risk evaluations of pesticides [10,114].

## 5. Conclusions

The analysis of a large set of pesticides (*n* = 338) belonging to various main chemical classes using an online tool *in silico* predicting human intestine and brain permeation indicated that a high percentage of pesticides (81.4%) was predicted to exhibit high intestinal absorption with a high accuracy (96%), whereas a lower, but substantial, percentage (38.5%) displayed brain permeation. Such data suggest that human exposure to pesticides through the oral way is likely to result in the intake of these environmental contaminants for most of them and brain permeation for some of them, thus supporting the well-established toxicity, including neurotoxicity, of pesticides for human health. They also highlight the interest of computational approaches for assessing pesticide pharmacokinetics and toxicity in humans.

## Figures and Tables

**Table 1:** Prediction of brain permeation for organophosphorus oxon metabolites and parent pesticide molecules ^1^.

Organophosphorus Pesticide	Brain Permeation Prediction	
	Parent Molecule	Oxon Metabolite
Bensulide	No	No
Chlorpyrifos	No	Yes
Chlorpyrifos-methyl	No	Yes
Coumaphos	No	No
Diazinon	No	No
Ethion	No	No
Fenthion	No	No
Fonofos	No	Yes
Malathion	No	No
Methyl-parathion	No	No
Parathion	No	No
Phorate	No	No
Phosmet	No	No
Sulprofos	No	No
Terbufos	No	No

^1^ Prediction was based on tPSA/WLogP-based graphical BOILED-Egg method.

**Table 2:** Physicochemical parameters of pesticides (*n* = 338) predicted to exhibit low or high intestinal absorption ^1^.

Physicochemical Parameter	Parameter Value (Mean ± SD)		
	Low Intestinal Absorption (*n* = 63 Pesticides)	High Intestinal Absorption (*n* = 275 Pesticides)	Significance ^2^
Molecular weight (g/mol)	420.4 ± 145.4	284.0 ± 79.3	S (*p* < 0.0001)
Mean atomic van der Waals volume	0.7 ± 0.1	0.6 ± 0.1	S (*p* < 0.0001)
Mean atomic polarizability	0.8 ± 0.5	0.7 ± 0.1	S (*p* < 0.0001)
Number of heavy atoms	24.2 ± 11.4	18.0 ± 5.3	S (*p* < 0.0001)
Number of aromatic heavy carbons	6.6 ± 6.3	5.5 ± 4.4	NS (*p* = 0.1050)
Fraction Csp3	0.5 ± 0.3	0.5 ±0.3	NS (*p* = 0.7335)
Number of rotatable bonds	6.2 ± 4.2	5.4 ± 2.2	S (*p* = 0.0252)
Number of H-bond acceptors	4.5 ± 4.3	3.5 ± 1.6	S (*p* = 0.0031)
Number of H-bond donors	0.4 ± 0.9	0.6 ± 0.8	NS (*p* = 0.0550)
LogS (Silicos-IT)	−6.0 ± 2.5	−3.8 ± 1.9	S (*p* < 0.0001)
XLogP3	5.2 ± 2.1	3.1 ± 1.8	S (*p* < 0.0001)
Molar refractivity	97.4 ± 37.6	73.2 ± 19.9	S (*p* < 0.0001)
tPSA (Å^2^)	68.1 ± 61.3	70.3 ± 27.7	NS (*p* = 0.6675)

^1^ Prediction was based on the tPSA/WLogP-based graphical BOILED-Egg method; ^2^ S, statistically significant between pesticides displaying predicted low or high intestinal absorption (*p* < 0.05); NS, not statistically significant (*p* ≥ 0.05).

**Table 3:** Physicochemical parameters of pesticides (*n* = 338) predicted to display brain or no brain permeation ^1^.

Physicochemical Parameter	Parameter Value (Mean ± SD)		
	No-Brain Permeation (*n* = 208 Pesticides)	Brain Permeation (*n* = 130 Pesticides)	Significance ^2^
Molecular weight (g/mol)	332.2 ± 120.2	273.1 ± 74.6	S (*p* < 0.0001)
Mean atomic van der Waals volume	0.7 ± 0.1	0.6 ± 0.1	S (*p* = 0.0225)
Mean atomic polarizability	0.7 ± 0.3	0.7 ± 0.1	NS (*p* = 0.0510)
Number of heavy atoms	20.0 ± 8.3	17.8 ± 4.8	S (*p* = 0.0061)
Number of aromatic heavy carbons	5.9 ± 5.2	5.4 ± 4.2	NS (*p* = 0.3634)
Fraction Csp3	0.5 ± 0.3	0.5 ± 0.3	NS (*p* = 0.4746)
Number of rotatable bonds	6.0 ± 2.9	4.8 ± 2.2	S (*p* < 0.0001)
Number of H-bond acceptors	4.3 ± 2.7	2.8 ± 1.3	S (*p* < 0.0001)
Number of H-bond donors	0.6 ± 0.9	0.6 ± 0.7	NS (*p* = 0.6422)
LogS (Silicos-IT)	−4.2 ± 2.5	−4.1 ± 1.6	NS (*p* = 0.6153)
XLogP3	3.6 ± 2.4	3.4 ± 1.4	NS (*p* = 0.4395)
Molar refractivity	81.1 ± 29.1	72.2 ± 18.4	S (*p* = 0.0021)
tPSA (Å^2^)	83.3 ± 39.2	48.5 ± 15.2	S (*p* < 0.0001)
Number of N and O atoms	4.4 ± 2.5	3.4 ± 1.2	S (*p* < 0.0001)

^1^ Prediction was based on the tPSA/WLogP-based graphical BOILED-Egg method; ^2^ S, statistically significant between predicted brain- and non-brain-permeant pesticides (*p* < 0.05); NS, not statistically significant (*p* ≥ 0.05).

**Table 4:** Comparison of measured and predicted human intestinal absorption for some pesticides.

Pesticide	Class	Intestinal Absorption ^1^	
		Determined from Pharmacokinetics Studies	Predicted by SwissADME Webtool
Cypermethrin	Pyrethroid	High (Fa = 0.40 [40,41])	High
Deltamethrin	Pyrethroid	High (Fa > 0.48 [42,43])	High
Permethrin	Pyrethroid	High (Fa ≥ 0.32 [44])	High
Bendiocarb	Carbamate	High (Fa ≥ 0.99 [45])	High
Pirimicarb	Carbamate	High (Fa = 0.74 [46])	High
Molinate	Carbamate	High (Fa > 0.40 [22])	High
Propoxur	Carbamate	High (Fa > 0.37 [47,48])	High
DDT ^2^	Organochlorine	Low (Fa = 0.15 [49])	Low
Pentachlorophenol	Organochlorine	High (Fa > 0.86 [50])	High
TCDD ^2^	Organochlorine	High (Fa > 0.87 [51])	Low
Chlorpyrifos	Organophosphorus compound	High (Fa = 0.82 [52,53])	High
Diazinon	Organosphosphorus compund	High (Fa > 0.66 [54])	High
Dichlorvos	Organophosphorus compound	High (Fa > 0.36 [55])	High
Dimethoate	Organosphosphorus compound	High (Fa = 0.86 [56,57])	High
Fenitrothion	Organophosphorus compound	High (Fa = 0.81 [58])	High
Parathion	Organophosphorus compound	High (Fa > 0.46 [59])	High
Propetamphos	Organophosphorus compound	High (Fa > 0.41 [60])	High
2,4,5-T ^2^	Miscellaneous	High (Fa > 0.89 [61])	High
2,4-D ^2^	Miscellaneous	High (Fa = 0.85 [62,63])	High
Fluazifop-butyl	Miscellaneous	High (Fa = 0.88 [64])	High
MCPA ^2^	Miscellaneous	High (Fa > 0.55 [65])	High
Paraquat	Miscellaneous	Low (Fa ≤ 0.05 [66])	Low
Picloram	Miscellaneous	High (Fa = 0.91 [67])	High
Triclopyr	Miscellaneous	High (Fa > 0.82 [68])	High
Warfarine	Miscellaneous	High (Fa > 0.93 [69])	High

^1^ Human intestinal absorption is considered high when Fa ≥0.30 and low when Fa <0.30; ^2^ DDT, dichlorodiphenyltrichloroethane; TCDD, 2,3,7,8-tetrachlorodibenzo-p-dioxin; 2,4,5-T, 2,4,5-trichlorophenoxyacetic acid; 2,4-D, 2,4-dichlorophenoxyacetic acid; MCPA, 2-methyl-4-chlorophenoxyacetic acid.

## Supplementary Materials

The following are available online at www.mdpi.com/1660-4601/14/7/708/s1, Figure S1: Comparison of physicochemical parameters of organochlorines (*n* = 30) predicted to display low or high intestinal absorption; Table S1: Pesticides-dataset; Table S2: Oxon organophosphorus pesticides-dataset.

## Appendix A

R code used for drawing ellipsoidal regions defining intestine and brain permeation according to the graphical BOILED-Egg method.

library("plotrix")myW = 1200

myH = 1000

ps = 6

jpeg(width = myW, height = myH, "ALL.05.png", res = "300", pointsize = ps)

dataAll = read.table("20170530_pesticides_swissadme.csv", sep = "t",header = T, dec = ",")

datasize = read.table("20170530_pesticides_swissadme.csv", sep = "t",header = T, dec = ",")

plot(datasize$TPSA, datasize$WLOGP, col=rgb(1,1,1), xlab = "tPSA (A²)", ylab = "WLog P")

draw.ellipse(71.051, 2.292, 142.081/2, 8.740/2, −1.031325, deg = TRUE, col = "grey")

draw.ellipse(38.117, 3.177, 82.061/2, 5.557/2, −0.171887, deg = TRUE, col = "yellow")

points(dataAll$TPSA, dataAll$WLOGP, cex=0.5)

draw.ellipse(71.051, 2.292, 142.081/2, 8.740/2, -1.031325, deg = TRUE)

dev.off()
