# Supplementary material for: In Silico Prediction for Intestinal Absorption and Brain Penetration of Chemical Pesticides in Humans
Source: Int J Environ Res Public Health. 2017 Jun 30;14(7):708. doi: 10.3390/ijerph14070708 (PMC5551146; doi:10.3390/ijerph14070708)
Supplement: Supplementary file 1 [file ijerph-14-00708-s001.zip › Figure S1.pdf]

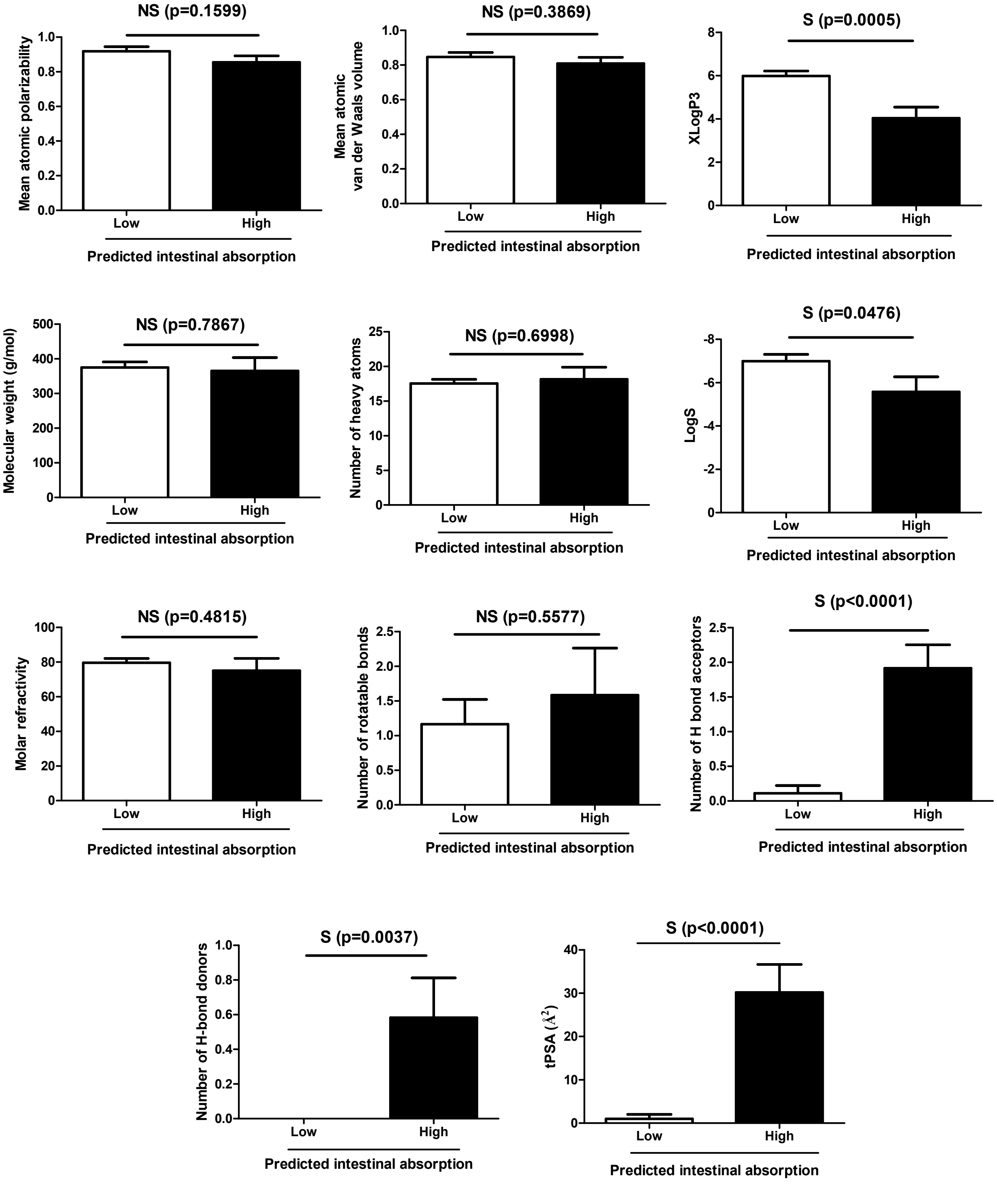

Figure S1: Physico-chemical parameters of organochlorines (n=30) predicted to display low or high intestinal absorption. S, statistically significant ($p<0.05$); NS, not statistically significant ($p>0.05$). Statistical analysis was performed with the unpaired Student's t test, excepted for the number of H-bond donors for which the Mann-Whitney test was used (due to the fact that unpaired Student's t test was not applicable because all the values for predicted low-permeant organochlorines are identical).
